# Shared decision-making quality and decisional regret in patients with low-risk superficial basal cell carcinoma: A prospective, multicenter cohort study

**DOI:** 10.1016/j.jdin.2023.05.015

**Published:** 2023-07-11

**Authors:** Andrea Catalan-Griffiths, Paola Pasquali, Salvador Arias-Santiago, Pedro Valeron, Antonio Martinez-Lopez, Maria Luz Negrin, Israel John Thuissard Vasallo, Cristina Andreu-Vazquez, Azael Freites-Martinez

**Affiliations:** a Dermatology Service, Medical University of Graz, Graz, Austria / AUVA Rehabilitation Clinic Tobelbad, Tobelbad, Austria; b Dermatology Service, Pius de Valls Hospital, Tarragona, Spain / Alcala University; c Dermatology Service, Virgen de las Nieves University Hospital, Granada, Spain; d Biosanitary Research Institute of Granada - ibs.GRANADA, Granada, Spain; e Dermatology Service, Dr Negrin University Hospital, Gran Canaria, Spain; f Santa Catalina Hospital, Gran Canaria, Spain; g Faculty of Biomedical Sciences and Health. Universidad Europea, Madrid, Spain; h Dermatology Service, Hospital Ruber Juan Bravo Hospital / Universidad Europea, Madrid, Spain

**Keywords:** superficial basal cell carcinoma, oncology, shared-decision making

## Abstract

**Background:**

Many therapies are available to treat low-risk superficial basal cell carcinoma (lr-sBCC), which may complicate the shared decision-making (SDM) process.

**Objective:**

To assess the SDM process of patients and physicians when deciding lr-sBCC therapy as well as the factors that may influence the SDM process.

**Methods:**

A prospective, multicenter cohort study was conducted over 18 months, from October 2018 to April 2020, in 3 tertiary university hospitals and 1 private hospital.

**Results:**

This study included 107 patients. There was a weak positive correlation between Shared Decision-Making Questionnaire-Patient version (SDM-Q-9) and Shared Decision-Making Questionnaire-Physician version (SDM-Q-Doc) (Spearman’s correlation coefficient [*r*_*s*_] [105] = 0.21; *P* = .03). Most patients (71%) chose a nonsurgical treatment after the SDM process. Patients with higher satisfaction with the SDM had lower decisional conflict and decisional regret (*P* < .001). Patients aged >80 years had higher rates of significant decisional conflict. When evaluating treatment decisions, the highest median score for decisional conflict (22, IQR [16]; *P* = .01) was observed among patients who chose a surgical excision.

**Limitations:**

Patients may have self-selected to participate.

**Conclusion:**

This study suggests that some patients may prefer less invasive therapies for lr-sBCC. The SDM process may reduce decisional conflict and decisional regret.

**Capsule Summary**

• Different treatments are available for low-risk, superficial basal cell carcinoma, which may hinder the patients’ decision regarding treatment.
• A higher agreement with the shared decision-making process reduces the patient’s decisional conflict and regret. A better understanding of the factors that influence this process may empower patient-physician communication and decisions.

## Introduction

The shared decision-making (SDM) model ensures that health care providers do not make decisions solely based on knowledge, experience, and scientific evidence as well-informed patients are encouraged to take an active part in their medical decisions.^1^ SDM is recommended to improve the quality of care of patients.^2^^,^^3^ However, there is limited data on SDM in dermatology.^4^

While surgical excision remains the gold standard for the management of low-risk superficial basal cell carcinoma (lr-sBCC), noninvasive techniques have become more widespread due to lower comorbidity and improved cosmetic outcomes.^3^ Giving clear information about these to the patients is also recommended,^4^ as any type of skin cancer may cause psychosocial distress.^5^ Therefore, patients with lr-sBCC may benefit from the SDM approach.^3^ This study aims to assess clinical and demographic factors that may influence the SDM process in patients with lr-sBCC and how it relates to decisional conflict and decisional regret.

## Methods

This was a prospective, multicenter cohort study conducted over 18 months, from October 2018 to April 2020. Patients were included from 4 dermatology services in Spain (3 university hospitals and 1 private hospital). Relevant clinical data were obtained from each electronic medical record. The study was approved by the review board of each participating institution.

The National Comprehensive Cancer Network guideline, version 1.2018, was used for the classification and management of lr-sBCC.^6^ The diagnosis was based on clinical and dermatoscopic features.^7^ Biopsies were obtained as needed.

### Participants

Adult patients with ≥1 lr-sBCC were invited to participate in this study. After receiving their consent, detailed review and explanation of all possible treatments (excisional surgery, Mohs micrographic surgery, cryosurgery, curettage and electrodesiccation [C&E], photodynamic therapy [PDT], and topical imiquimod [5%]) were given using a visual SDM-decisional aid tool. Participating dermatologists received previous training on SDM.

#### Questionnaires

The Shared Decision-Making Questionnaire-Patient version (SDM-Q-9) assesses the patient’s perception of the SDM process.^8^ The scores range from 0 (no SDM) to 100 (high degree of SDM). The Shared Decision-Making Questionnaire-Physician version (SDM-Q-Doc) evaluates the physician’s perceptions of SDM, and this questionnaire is structured and scored similarly to SDM-Q-9.^9^ The Decisional Conflict Scale (DCS) determines the patient’s uncertainty about the treatment decision.^10^ A total DCS score of ≥25 suggests clinically significant decisional conflict. The Decisional Regret Scale (DRS) assesses regret about treatment decisions. Scores of ≥26 indicate strong decisional regret.^11^ All questionnaires are validated in Spanish.

### Procedure

Patients completed the SDM-Q-9 and DCS at the first consultation. They completed DRS 6 months after the treatment via a telephone call. The dermatologists completed the SDM-Q-Doc immediately after the SDM process.

#### Statistical analysis

The data analysis was conducted with SPSS, version 27.0 (IBM Corp), with a significance level of 5%. Descriptive analysis of qualitative variables was performed, while quantitative variables without normal distribution were expressed as median and IQR. When assessing DCS on a qualitative basis, χ^2^ test was used, while Kruskal-Wallis and Mann-Whitney tests were used for its score evaluation based on the treatment decision and tumor location, respectively.

## Results

A total of 107 patients, with 50 women and 57 men, were included, with a mean age (SD) of 69 (14) years. Most patients (71%) chose a nonsurgical treatment for their lr-sBCC treatment (cryosurgery, C&E, imiquimod, or PDT), and cryosurgery was the most selected treatment (37%) (Table I).

**Table I:** Baseline characteristics of patients (*N* = 107)

Age	*n* (%)
Mean ± SD	69 ± 14
Age group	
<60	23 (22)
61–80	59 (55)
>80	25 (23)
Sex	
Male	57 (53)
Female	50 (47)
Marital status	
Single	19 (18)
Widow	22 (21)
Married	66 (62)
Education	
Undergraduate	65 (61)
Graduate	42 (39)
Income/y	
≤€30.000	80 (75)
>€30.000	27 (25)
Treatment costs	
Social or private insurance	84 (79)
Out-of-pocket	23 (21)
Superficial lr-BCC as reason of consultation	
No	33 (31)
Yes	74 (69)
Tumor’s location	
Head + neck	57 (53)
Extremities + trunk	50 (47)
Tumor’s size	
<0.5 cm	41 (38)
0.5–2.0 cm	66 (62)
Selected treatment after SDM	
Cryosurgery	40 (37)
Surgery	31 (29)
Curettage and electrodessication	15 (14)
Imiquimod	14 (13)
Photodynamic therapy	7 (7)

Means and SDs are reported for continuous variables.

*lr-BCC*, Low-risk basal cell carcinoma; *NMSC*, nonmelanoma skin cancer; *SDM*, Shared Decision-Making.

**Fig 1:**
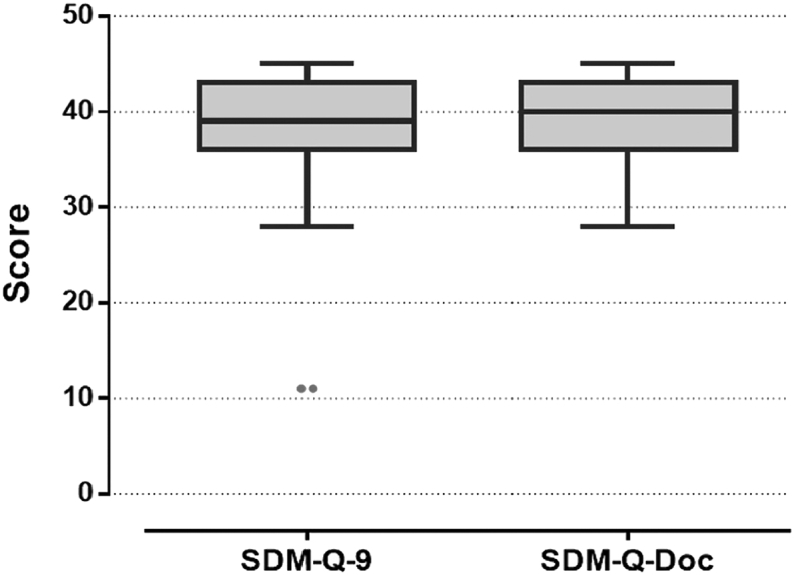
Self-reported shared decisional conflict in patients and physicians. The middle line represents the median score on each questionnaire. The upper and lower bars represent the 25^th^ and 75^th^ centiles, respectively. The dots represent 2 outliners. *SDM-Q-9*, Shared Decision-Making Questionnaire-Patient version; *SDM-Q-Doc*, Shared Decision-Making Questionnaire-Physician version.

**Fig 2:**
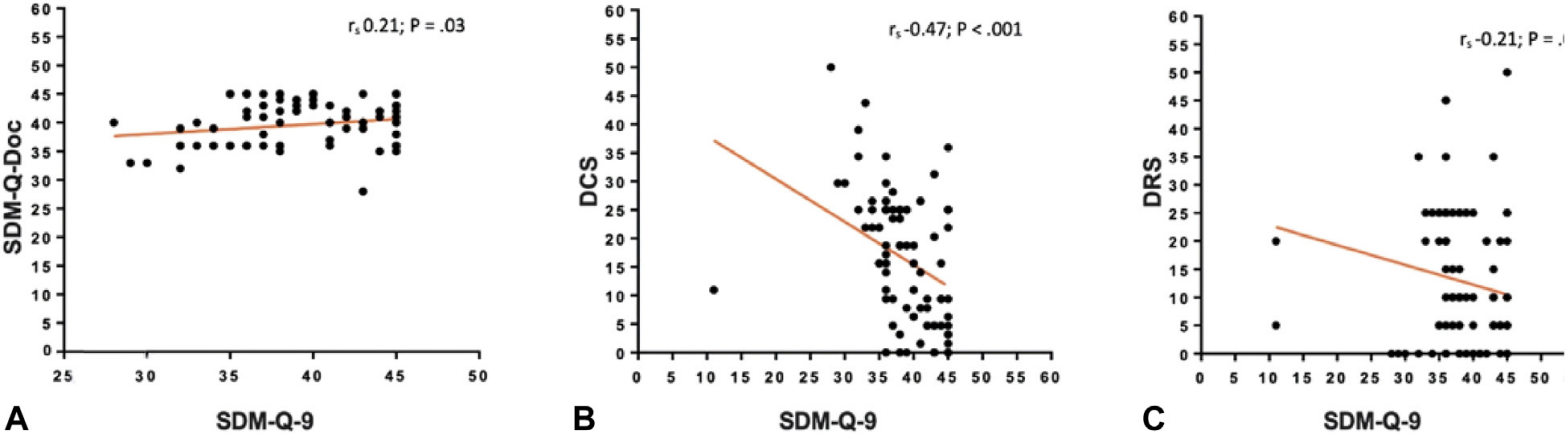
Scatter plot of the Spearman’s correlation between scores. **A,** SDM-Q-9 and SDM-Q-Doc, **B,** DCS and SDM-Q-9, and **C,** DRS and SDM-Q-9. *r*_*s*_, Spearman’s correlation coefficient; *SDM-Q-Doc*, Shared Decision-Making Questionnaire-Physician version; *SDM-Q-9*, Shared Decision-Making Questionnaire-Patient version; *DCS*, Decisional Conflict Scale; *DRS*, Decisional Regret Scale.

The internal consistency (Cronbach α) of the SDM-Q-9 and SDM-Q-Doc was 0.93 and 0.88 respectively, indicating an excellent scale reliability. Median SDM-Q-9 score was 38, IQR [7], and median SDM-Q-Doc was 40, IQR [7] (Fig 1). Spearman’s correlation between these questionnaires was *r*_*s*_ [105] = 0.21; 95% CI, 0.01 to 0.39; *P* = .03, indicating a weak positive correlation (Fig 2).

### Decisional conflict

The DCS had a good scale reliability (Cronbach α of 0.89). Forty patients (37%) experienced significant decisional conflict (DCS ≥ 25). Higher rates of significant conflict were found in patients aged ≥80 years (56%) vs 22% in patients aged <60 years (*P* = .04) (Supplementary Table I, available via Mendeley at https://data.mendeley.com/datasets/h6y6nhwdfy/5). Moreover, higher rates of significant conflict were found among widowed patients (59%; *P* = .02) when compared to patients with other marital status (Supplementary Table II, available via Mendeley at https://data.mendeley.com/datasets/h6y6nhwdfy/5). The highest median decisional conflict was found among patients who chose surgical excision and the lowest among those who chose C&E (Supplementary Table III, available via Mendeley at https://data.mendeley.com/datasets/h6y6nhwdfy/5). Patients with lr-sBCC located on the head and neck areas also had higher median scores of decisional conflict when compared to patients with tumors on other locations (Supplementary Table IV, available via Mendeley at https://data.mendeley.com/datasets/h6y6nhwdfy/5). Patients with higher SDM reported lower decisional conflict, with a moderate negative correlation (DCS and SDM-Q-9 *r*_*s*_ [107] = −0.47; 95% CI, −0.61 to −0.30; *P* < .001) (Fig 2).

The Cronbach α for the DRS was 0.86, indicating good reliability. Only 9 patients (8%) reported strong decisional regret (DRS ≥ 26). A weak negative correlation was found between the DRS and SDM-Q-9 scores (*r*_*s*_ [107] = −0.21; 95% CI, −0.39 to −0.02; *P* = .02) (Fig 2). From the 9 patients who reported significant decisional regret (DRS ≥ 26), 5 were aged between 61 and 80 years, and 4 were older than 80 years (all widows). Six of them had lr-sBCC located on the head, 2 on the trunk, and 1 on the upper extremities. Four of them chose surgical excision, 3 chose cryosurgery, 1 chose PDT, and 1 chose C&E.

## Discussion

In this study, patients and physicians had a tendency to agree on the SDM process for the treatment of lr-sBCC. A higher score of decisional conflict was found in patients who chose a surgical treatment or in those with tumors located on the head and neck area. Scarring after skin cancer surgery may change physical appearance and negatively impact psychosocial functioning and treatment decision.^12^ Pretreatment assessment and SDM process may help to identify patients with scar concerns to offer appropriate counseling and support.^13^

Patients aged >80 years reported higher rates of significant decisional conflict when compared to younger patients. Although they agreed to participate in this study, we may speculate that older patients are used to the paternalistic medical model (physicians decide the treatment) or may be overwhelmed with the amount of treatment information given during the SDM.

The clinical implementation of a controlled SDM process in patients with lr-sBCC and other dermatologic conditions may be beneficial.^14^ We found that patients with lower decisional conflict and decisional regret tended to have higher agreement with the SDM; such findings were also reported recently in patients with alopecia areata.^15^ In addition, the SDM model has been used in some medical conditions as a presumption of informed consent when a validated decision aid is used in the process.^16^

Some limitations should be recognized in this study. First, there was a possible cofounding factor when evaluating decisional conflict in widows, who also corresponded to being older patients. Second, although participating dermatologists underwent the same SDM training to take part in the study, individual treatment preferences may have influenced the patient’s perception. Third, patients may have self-selected to participate. Lastly, there was a lack of histopathologic confirmation of lr-sBCC as the study was based on dermatoscopic findings. Despite these limitations, to the best of our knowledge, this is the first study assessing the SDM process on patients with lr-sBCC.

SDM and patient-centered care should be especially important as different treatment options for lr-sBCC are approved and recommended by different medical guidelines with similar outcomes that may cause difficult treatment decisions.^6^ Furthermore, our study shows that some sociodemographic and clinical factors may pose challenges in patient-physician communication and treatment decisions.

It is important to raise awareness among dermatologists about the benefits of the SDM on lr-sBCC and the need of effectively communicating with the patient in order to prevent decisional conflict and decisional regret. A better understanding of this process in different clinical settings may represent an opportunity to empower communication and satisfaction between patients and dermatologists.

## Conflicts of interest

AC-G, PP, SA-S, PV, AM-L, MLN, ITV, and CA-V have nothing to disclose. AF-M is consultant of ISDIN, L’Oreal, Galderma, and Shook, Hardy, Bacon LLP who represent Sanofi Aventis US LLC
